# Surgical Innovations in Tracheal Reconstruction: A Review on Synthetic Material Fabrication

**DOI:** 10.3390/medicina60010040

**Published:** 2023-12-25

**Authors:** Usman Khalid, Petar Uchikov, Bozhidar Hristov, Krasimir Kraev, Maria Koleva-Ivanova, Maria Kraeva, Atanas Batashki, Daniela Taneva, Mladen Doykov, Angel Uchikov

**Affiliations:** 1 Medical Faculty, Medical University of Plovdiv, 4000 Plovdiv, Bulgaria; usmankhalid957@gmail.com; 2 Department of Special Surgery, Faculty of Medicine, Medical University of Plovdiv, 4000 Plovdiv, Bulgaria; 3 Section “Gastroenterology”, Second Department of Internal Diseases, Medical Faculty, Medical University of Plovdiv, 4000 Plovdiv, Bulgaria; 4 Department of Propedeutics of Internal Diseases, Medical Faculty, Medical University of Plovdiv, 4000 Plovdiv, Bulgaria; 5 Department of General and Clinical Pathology, Faculty of Medicine, Medical University of Plovdiv, 4000 Plovdiv, Bulgaria; 6 Department of Otorhynolaryngology, Medical Faculty, Medical University of Plovdiv, 4000 Plovdiv, Bulgaria; 7 Department of Nursing Care, Faculty of Public Health, Medical University of Plovdiv, 4000 Plovdiv, Bulgaria; 8 Department of Urology and General Medicine, Medical Faculty, Medical University of Plovdiv, 4000 Plovdiv, Bulgaria; mladen.doykov@mu-plovdiv.bg

**Keywords:** tracheal reconstruction, 3D printing, tissue engineering

## Abstract

*Background and Objectives*: The aim of this review is to explore the recent surgical innovations in tracheal reconstruction by evaluating the uses of synthetic material fabrication when dealing with tracheomalacia or stenotic pathologies, then discussing the challenges holding back these innovations. *Materials and Methods*: A targeted non-systematic review of published literature relating to tracheal reconstruction was performed within the PubMed database to help identify how synthetic materials are utilised to innovate tracheal reconstruction. *Results*: The advancements in 3D printing to aid synthetic material fabrication have unveiled promising alternatives to conventional approaches. Achieving successful tracheal reconstruction through this technology demands that the 3D models exhibit biocompatibility with neighbouring tracheal elements by encompassing vasculature, chondral foundation, and immunocompatibility. Tracheal reconstruction has employed grafts and scaffolds, showing a promising beginning in vivo. Concurrently, the integration of resorbable models and stem cell therapy serves to underscore their viability and application in the context of tracheal pathologies. Despite this, certain barriers hinder its advancement in surgery. The intricate tracheal structure has posed a challenge for researchers seeking novel approaches to support its growth and regeneration. *Conclusions*: The potential of synthetic material fabrication has shown promising outcomes in initial studies involving smaller animals. Yet, to fully realise the applicability of these innovative developments, research must progress toward clinical trials. These trials would ascertain the anatomical and physiological effects on the human body, enabling a thorough evaluation of post-operative outcomes and any potential complications linked to the materials or cells implanted in the trachea.

## 1. Introduction

The trachea is a U-shaped semiflexible tube measuring a length of 10–13 cm in length, consisting of incomplete rings with hyaline cartilage on anterior and lateral walls and the posterior border layered by trachealis smooth muscle. Beginning inferiorly from the larynx, it extends from C4–C5 to T4–T5, terminating at the tracheal carina before dividing into the left and right main bronchus [1,2]. The functional physiology of the trachea facilitates crucial roles by providing air conduction between the larynx and bronchi, enabling heat and moisture exchange and particle elimination [2]. Tracheal abnormalities are often divided into two categories: congenital and acquired. Congenital tracheomalacia and tracheal stenosis are more commonly associated with infants [3], whereas acquired tracheal lesions are associated with malignancy, traumatic injury, complications of tracheostomy tubes, and subglottic stenosis [4]. Tracheomalacia and stenosis are fatal conditions for adults and infants, necessitating appropriate intervention according to the severity [5]. CPAP through tracheostomy was shown to aid infants with moderate to severe tracheomalacia, but surgery is more often indicated for adults with severe cases of tracheomalacia [6,7]. When evaluating a surgical approach to dealing with tracheal disorders, resection and anastomosis are specified for segment stenosis with a length of 4–5 cm or less in adults and 2 cm or less in children [8].

With the innovative advancements in synthetic material fabrication, a promising new avenue has emerged as a potential alternative to conventional approaches (Table 1). Successful tracheal reconstruction using such technology requires 3D models to have a biocompatible nature with the adjacent tracheal components including vasculature, chondral foundation, all the way down to a histological cellular level [9]. Despite the development of advanced techniques and new source materials, the most complex challenges lie in the complexity of the tracheal anatomy, due to its limited blood supply and its cylindrical framework allowing longitudinal flexibility and lateral stability.

## 2. Methods

A targeted non-systematic review of published literature relating to tracheal reconstruction was performed with PubMed and Google Scholar databases scouted to help provide a more defined understanding on the recent innovations regarding 3D printing and bioengineering and their impact on the future of tracheal reconstruction. Different combinations of keywords and phrases were used to narrow down and establish relevant source material. Keywords included, but were not limited to, tracheal reconstruction, 3D, three-dimensional printing, tissue engineering, tracheomalacia, bioinks, tracheal grafting, bio gels, tracheal resorbing models, and stem cells. All material published before 30th October 2023 was eligible for inclusion in this review. Articles that were not published in English were not included in this literature review. Articles were included if deemed relevant and included information associated to the keywords.

## 3. Results

### 3.1. Tracheal Anatomy

To successfully perform surgical manoeuvres on the trachea, an established understanding of tracheal blood supply is required alongside the positional relationship between the vasculatures. This is vital when avoiding sequalae of tracheal ischemia. The tracheal blood supply is divided into upper (cervical) and lower (thoracic) with the upper being supplied by the inferior thyroid artery whilst the lower tracheal supply is through the first tracheoesophageal arteries and bronchial arteries directly from the aorta. The lateralised location of the arteries along the longitudinal section must be considered. The anteromedial aspect of the descending aorta gives rise to the superior bronchial artery, which is lateral to the carina and posterior to the left main bronchus. The anterior branch of the bronchial artery supplying the anterior carina courses over the proximal left main stem bronchus [10]. The structural relationship is important to bear in mind during surgery, with nearby structures at higher risk of iatrogenic injury. With the trachea, the left and right thyroid lobes sit anterolateral to the upper trachea. With the oesophagus beginning at the level of the cricoid cartilage, it passes along the left posterior border of the trachea. The right posterior border of the trachea is positioned anteriorly to the anterior surface of the vertebral bones. During the dissection of the proximal trachea, nerve orientation is key to preserve neurovascular structures. The recurrent laryngeal nerves enter the larynx between the thyroid and cricoid cartilage under the inferior horn of the thyroid cartilage. The left recurrent laryngeal nerve originates beyond the aortic arch, then curves posteriorly, running just lateral to the ligamentum arteriosum. It subsequently loops back and ascends along the left tracheoesophageal groove towards the cricoid cartilage. The right recurrent laryngeal nerve branches from the right vagus nerve shortly after the right subclavian artery, diving posteriorly beneath its origin. It then recurs and ascends within the right tracheoesophageal groove toward the cricoid cartilage. Damage to the recurrent laryngeal nerves, whether due to complete or partial transection, ischemia, contusion, traction thermal injury, or tumour intrusion, can lead to vocal cord weakness or complete paralysis. This may result in hoarseness or even complete loss of voice or airway function, depending on the severity of the injury and the condition of the opposite nerve. Surgeons operating on the proximal trachea must be cautious and mindful of these nerves’ paths during dissection [11].

### 3.2. Aliphatic Polymers

The potential personalising ability of scaffolded materiel could provide major benefits with enhanced mechanical strength and excellent biocompatibility [12]. An increase in material availability with 3D bioprinting provides a greater variety in material durability and compatibility [13]. When evaluating biomaterials, it is key for them to have similar anatomical and physiological properties to the tracheal cartilage. A group of aliphatic polymers including poly-caprolactone (PCL), poly-glycolic acid (PGA), poly-lactic acid (PLA), and poly-lactic-co-glycolic acid (PLGA) have been extensively researched in their potential application and integration in tracheal reconstruction through 3D printing.

### 3.3. Tracheal Grafting and Scaffolding

PCL is at the forefront of tracheal biomaterial research, providing superb results in maintaining airway patency, tracheal wall integration, granulation tissue prevention, and involvement in mucosalisation in respiratory epithelium when used to recreate a partial tracheal defect [14]. Using an electrospun patch, including randomly layered PCL nanofibers enveloping 3D printed PCL rings, an airtight, bio-resorbable graft was created and tested for airway support in sheep with all surviving for at least 10 weeks [15]. When used in rabbits, none showed signs of respiratory distress and electron microscopy confirmed the regeneration of cilia with its beating frequency identical to natural tracheal cilia [16]. The challenge of vascularisation was approached with a PCL/vacuum-assisted decellularised trachea, which exhibited angiogenesis in vivo alongside low immunogenicity [17]. It has an ideal non-toxic degradation alongside low porosity, which stimulates chondrogenesis, thus strengthening the cartilage [18]. All this, coupled with its low boiling point, allows PCL to be easily printed with 3D rendering machines, making its use economically viable [19]. PGA and PLA were trialled in laryngotracheal reconstruction in rabbits with results showing stable airtight airways and re-epithelialisation seen alongside minimal scarring and no stenosis, although its stability in larger animals has yet to be seen [20]. A unique yet innovative approach was taken by Kang et al. when incorporating electrospun PLA membranes that enveloped a 3D printed (3DP) thermoplastic polyurethane skeleton. With this, an antibacterial enhancement was made with the introduction of ionic liquid functioned graphene oxide. In vivo results indicated that the scaffolds possessed strong biocompatibility with the results showing no inflammation, infection, or interfacial transition zone between the electrospun membrane and subcutaneous tissue. In vitro results demonstrated the regenerative properties of the scaffold, thus exemplifying the desirability of these graft materiel combinations for tracheal reconstruction [21].

The integration of 3D printed PCL microfibres with an artificially designed trachea was shown to enhance tracheal cartilage and mucosa regeneration through the combination of human induced pluripotent derived stem cells and a two-layer tubular layer scaffold. Micro-CT analysis showed effective neocartilage formation at defect sites [22]. When a 3D printed (3DP) PCL scaffold was coated in mesenchymal stem cells (MSC’s) seeded in fibrin, the half-pipe tracheal graft was able to restore shape and function in rabbits without any graft rejection [16]. Similarly, when 3DP poly-L-Lactic acid scaffolds were seeded with chondrocytes in rabbits via end-to-end anastomosis, there was successful tracheal segment regeneration alongside neo-angiogenesis, which facilitated the survival of the chondrocytes [23]. With thermoplastic polymers being ideal due to their boiling points and cost of printing, a blend of thermoplastic polyurethane and PLA have ideal properties due to their biocompatibility and absorption/degradation rate [24]. When the trachea is based on a 3DP scaffold, it acts as the baseline foundation, but when regenerative stem cells were incorporated into the scaffold, it transforms its applicational ability due to its implantable nature allowing it to carry out the anatomo-physiological function while regenerating the trachea and adapting to the surrounding tracheal structures. In an independent study, porcine derived small intestine submucous extracellular matrix patches were wrapped around PCL supports and lined the inside and outside of the graft, which allowed the animals to remain clinically stable for two weeks with no respiratory distress [25]. Park et al. went a step further by characterising PCL scaffolds 3D printed by the 4-axis fused deposition modelling method to determine any additive properties by changing the method of 3DP. The 4-axis fusion deposition model provided a greater dimensional accuracy relative to a scaffold and with the pre-defined pore size and pore interconnectivity being significantly closer to CAD software designs. Furthermore, the tensile and compressive strength of the 4-axis scaffold was mechanically superior to conventional scaffolding thus imitating tracheal tissue with pinpoint accuracy [26]. A combination of tissue engineering and cryopreservation was used to fabricate a neo trachea using pre-epithelialised cryopreserved tracheal allograft (ReCTA). Cryopreservation enables the long-term survival of an allograft alongside a reduction in antigenicity. ReCTA assisted in fibrosis obliteration and maintained airway patency in the neo-trachea that, when pedicled with an adipose flap, can be easily incorporated into the tracheal construct to allow neovascularisation [27].

### 3.4. Resorbing Models

In a patient with paratracheal tumour resection alongside tracheal reconstruction, a resorbing synthetic mesh was successfully placed with a myocutaneous pedicled pectoralis flap. After a 9-month follow-up, the patient showed a well-healed flap with no evidence of tracheomalacia, and no breathing complaints 12 months after the procedure [28]. Tian et al. has previously incorporated 3DP to produce a custom titanium mesh for patients with thyroid cartilage reconstruction [29]. A similar concept can be applied to the use of a nickel-titanium shape memory alloy mesh, which produced promising results in patients, where a woman with left vocal fold paralysis and subglottic atresia had the memory alloy mesh surgically implanted as an extraluminal tracheal scaffold and showed no signs of dyspnoea or discomfort upon exertion [30]. Marlex mesh has been used in conjunction with pericardium to manipulate tracheal reconstruction in 13 patients suffering from malignancy. With the results of the mesh insertion being successful, expansion on its use can be trialled [31]. Given the polypropylene foundation of a Marlex mesh [32], its potential for 3DP holds major value for the future of mesh-based tracheal reconstruction.

Autologous tracheal implants supported by silicone stents using endothelial cell spherules were evaluated by Taniguchi et al. with successful implantation alongside histological chondrogenesis and vasculogenesis [33]. PLA-based external splint rings were placed ex vivo in porcine tracheas that demonstrated a reduction in airway collapse [34]. Further research into long tracheal segment defects performed by Lumei Liu et al. tested composite tracheal grafts composed of 3DP splints with partially decellularised tracheal grafts, with the splint designed to deliver consistent mechanical support throughout the 3-month experimental period. Long term in vivo effects displayed no vascular disruption, anastomotic injury, or airway injury with no evidence of chronic inflammation, reinforcing its biocompatible nature [35]. A PCL based splint was implemented into a paediatric patient with tracheobronchomalacia, which resulted in relief of the life-threatening malacic condition by restoring airway patency [36]. Having successfully treated five children, Zopf et al. hope to launch an FDA clinical trial with eventual FDA approval [37]. Greene et al. expanded the repertoire through the innovative application of 3DP PCL stents in a patient with Floyd Type 1 tracheal agenesis, which facilitated the survival for the patient through its airway support [38]. A unique patient with combined congenital heart disease and tracheomalacia showed no airway stenosis when implanted with an external PCL splint, highlighting its safety and efficacy in patients with mixed pathologies [39]. Javia and Zur emphasised the use of microplate buttressing in open-airway reconstruction by successfully performing laryngotracheal reconstructions on seven children with no complications [40]. Gostridi et al. evaluated the efficacy of extraluminal bioresorbable plates to treat refractory localised airway malacia and ruled it as a valid therapeutic option in the treatment of upper airway malacia with some cases showing complete resolution of tracheomalacia allowing for a rapid decannulation proceeding plate implantation [41]. Microplate therapy was utilised in three patients with severe suprastomal collapse, with all patients decannulated without any postoperative complications [42].

### 3.5. Stem-Cell Biology and Epithelial Reconstruction

The continuity of the tracheal epithelium and mucosa is breached in congenital tracheal anomalies, which compromises the structure and functionality. With synthetic and allogeneic materials studied, a reliable tracheal tissue replacement is missing, fuelling expanded research into tissue engineering. By isolation, respiratory epithelial cells from the nasal turbinate are incorporated into calcium chloride polymerised blood plasma to produce human tissue respiratory epithelial construct (HTREC). After gene expression analysis, Ki67 (a proliferative marker) and MUC5AC (a mucin-secreting marker) both had significant increases in gene expression levels after four days, highlighting the viability of HTREC as an option for respiratory epithelial reconstruction [43]. When autologous nasal epithelial sheets were incorporated into a partially decellularised scaffold in rabbits, the cell sheets enhanced epithelial regeneration and the transplanted patches were wholly incorporated into the trachea after two months [44]. Yang et al. conceptualised a scaffold-free strategy, incorporating cartilage sheets reshaped to construct a cartilage tube embedded with an epithelial sheet. This scaffold-free trachea established a healthy blood supply through heterotropic vascularisation and showed promising mechanical properties due to its matrix-rich cartilage structure. With the epithelial foundation, the model displayed beneficial ciliary differentiation capabilities. A combination of such characteristics demonstrates its clinical potential for long-segment tracheal reconstruction [45]. The 90-day mortality rate was low among patients who underwent airway bioengineering using cryopreserved aortic matrices. Immunodetection and engraftment studies exhibited successful results showing de novo cartilage generation within the aortic allograft. The matrices stimulated proangiogenic, proinflammatory, chemoattractant, and immunomodulatory cytokines, which reinforces the hypothesis of the aortic matrices stimulating stem cell homing allowing de novo cartilage formation [46]. Martinod et al. followed up by reporting that most patients were breathing and speaking normally without the requirement of a tracheostomy or a stent at a long term follow up [47].

Another study by Martinod E, Radu DM, Onorati I et al. is TRITON-01, which evaluates the viability of airway bioengineering using stented aortic matrices as a standard procedure for airway replacement. Over a period of 12 years, 35 patients underwent airway replacement for either malignant or benign conditions. Results indicated a 2.9% mortality rate and a 22.9% morbidity rate within 30 days post-surgery. At a median follow-up of 29.5 months, 27 patients were alive, with no deaths directly related to the implanted bioprosthesis. However, stent-related granulomas were observed in 52.9% of patients requiring treatment. Only 28.6% achieved a stent-free survival, but the overall 2- and 5-year survival rates were encouraging at 88% and 75%, respectively. The study confirms the feasibility of using stented aortic matrices for airway replacement, suggesting its potential as standard care [48].

In a study looking at patients with tracheomalacia due to mustard gas exposure, MSC’s demonstrate anti-inflammatory properties, which act against the chronic inflammation caused by the gas. To counter the problems of vascularisation, the incorporation of angiogenic factors such as vascular endothelial growth factor, platelet-driven growth factor, and basic fibroblast growth factor can be deployed for angiogenic expansion [49]. In a polypropylene mesh tube with an atelocollagen layer, the collagen soaked in bone marrow MSC’s produced reduced stenosis and contributed to rapid epithelialization when compared to a soak in peripheral blood [50]. Another study expressing the plausibility of porcine cartilage powder seeded with MSC’s for tracheal repair defects was investigated by Shin et al., with none of the six rabbits showing signs of postoperative respiratory distress. Further endoscopic examination revealed tracheal regeneration with respiratory epithelium with no collapse or blockage. A ciliary beating frequency measurement of the regenerated respiratory epithelium was taken after 10 weeks and was deemed to have no statistical difference from normal epithelium [51]. Similar results were documented when the porcine cartilage was seeded in a chondrocyte medium, reinforcing the impact of a scaffold-seed composite [52]. The involvement of induced Pluripotent stem cells (iPs) offers a fresh perspective on tracheal regeneration. When evaluating ex vivo tracheal repair using human iPs derived silk fibroin collagen vitrigel membrane patches, evidence of mucociliary epithelium maintenance was observed alongside native tracheal epithelial metabolism [53]. Motile cilia were also observed in rats following histological analysis two weeks after the iPs based collagen scaffold was introduced as a tracheal segment [54]. Kim et al. studied the impacts of a multi-seed complex with a tracheal graft combined with iPs cell derived MSCs and chondrocytes. The artificially designed tracheas were successfully transplanted into a rabbit model with a segmental tracheal defect. Staining methods evinced a notable formation of cartilage in both the MSC and chondrocyte-based tracheal models in conjunction with mucociliary regeneration four weeks following implantation. A combination of cell seeds provides a promising outlook in the innovations of tracheal regeneration [22].

Research into the stem cell content of skin epithelial cells was performed due to difficulty obtaining cells from the trachea, mainly to do with its position and limited accessibility. After implanting the skin epithelial stem cells, within five months the tracheal epithelium was completely covered by ciliated cells despite the skin and tracheal epithelium originating from different germinal layers. Beyond their survival, they transdifferentiated into tracheal epithelial cells and chondrocytes verified through fluorescent cell analysis using a PKH26 dye hence accentuating the possibility of their use in transplantational reconstruction of the trachea [55]. Another study carried out by Gao et al. explored the protection of the tracheal cartilage by the skin derived epithelial lining, which assisted in facilitating the orthotropic tracheal transplantation and inhibiting tracheal hyperplasia. This combination aided the airway patency and survival rates of the rabbits tested [56].

### 3.6. Bioink Usage for Structural Biomimicking

Through the utilisation of photocrosslinkable tissue specific bioinks, a novel strategy was developed, incorporating 3DP to produce a cartilage vascularised fibrous tissue integrated trachea. The physiological importance of maintaining the tracheal architecture assists in mimicking tracheal function. Sun et al. previously used an O-shaped tracheal design but, to comply with functionality, a C shaped biomimetic 3DP trachea was manufactured and its mechanical adaptability and physiological regeneration tested. The C shape effectively dissipated anisotropic forces, thereby enabling a natural dynamic movement of the trachea. Cytocompatibility testing of the GC-Gel and GE-Gel showed no cytotoxicity reinforcing the photocrosslinkable nature of these hydrogels. A 19-min print time eases any trouble of inefficiency with the printing having no impact on cell viability [57]. Huo et al. expanded on this concept by placing the 3DP cartilage vascularised fibrous tissue integrated trachea in a rabbit model wrapped by a vascular muscle flap. A chondrocyte loaded photocrosslinkable cartilage specific bioink was used to create the C rings and the vascularised fibrous ring was constructed by a vascularised fibrous tissue specific bioink. After eight weeks, the rabbits trachea exhibited a complete and continuous tubular structure with a smooth inner surface and a satisfactory integration into the native trachea [58]. Using a multichannel coaxial extrusional systems for microfluidic bioprinting of multilayered tubular tissues, bioinks consisting of gelatin methacrolyl, alginate, and eight arm poly (ethylene glycol) acrylate with a tripentaerythritol core were used to construct several tubular tissues with adequate differentiation, proliferation, and cell viability, thereby demonstrating its capability to create tubular architecture [59].

### 3.7. Material Summary

Given the variety of scaffold materials that have been discussed in the review, we have included a summary of the materials with their individual analysis to provide a clear and distinguished outlook on each one to better assess their uses.

**Table 1:** Summary of synthetic materials used for tracheal reconstruction.

Type of Material	Fabrication	Degradation	Biocompatibility	Mechanical Properties	Study Type	Surgery Type	Outcome
Poly-caprolactone (PCL) [12,14,15,36,38]	1. Patch 2. Splint 3. Stent	2–3 years	1. Nontoxic 2. Maintains Airway patency 3. Compatible integration into tracheal wall 4. Minimal granulation tissue formation 5. Enables mucosalization with respiratory epithelium	1. Low melting point for shaping 2. Good flexibility at room temperature 3. Semi—crystalline polymer	Patch—In Vivo with Sheep Splint—Clinical Trial with paediatric patient Stent—Clinical Trial with paediatric patient	Patch—Patch tracheoplasty Splint—Bronchoplasty Stent— Esophagotracheoplasty	Patch—Sheep survived up to 10 weeks. Splint—Patient survival Stent—Patient survival
Poly-glycolic acid (PGA) [12,20]	Mini plate	6–12 months	1. Nontoxic 2. Maintains airway patency 3. Complete resorption and integration into tracheal wall 4. Good re-epithelialization with some rabbits exhibiting neocartilage formation 5. Some rabbits exhibited mild inflammation focussed on the plate implant.	1. Rigid with a poor flexibility 2. Semi—crystalline polymer	In vivo with Rabbits	Laryngotracheoplasty	All rabbits survived.
Poly-lactic acid (PLA) [12,24]	Splint	1–2 years	1. Nontoxic 2. Good biocompatibility and integration into the tracheal wall. 3. Demonstrated high water absorption like regenerative process of native tissue 4. Maintenance of airway patency	1. Semi—crystalline polymer 2. Does not exhibit an elastic nature which makes it inflexible	Ex Vivo	No surgical intervention as study was performed Ex Vivo	The model demonstrated that tracheomalacia can be successfully treated with a PLA based 3DP splint.
Poly-lactic-co-glycolic acid (PLGA) [12,40]	Microplate	1–2 years	1. Low toxicity if resorbed below 2 years 2. Can induce an acute and chronic inflammatory response which could lead to scarring and fibrous capsule formation 3. Maintains airway patency	1. Has a high tensile strength 2. Has a high flexural and bending strength. 3. Low melting point for shaping	Clinical Trial with paediatric patients	Microplate partial tracheoplasty	All patients survived.
Nickel-titanium alloy [12,30]	Mesh	Does not degrade	1. Can be toxic at high levels 2. Alloy has a high biocompatibility with human tissue 2. Small granulomas from chronic inflammation but did not impede airflow	1. Shape memory alloy 2. Woven mesh reinforced fibrous tissue to increase strength.	In Vivo with Dogs Clinical trial with one patient.	Mesh partial tracheoplasty	1 dog died 5 days after the operation and the remaining survived during the observation period. Patient in a clinical trial survived.
Polypropylene [12,49]	Mesh	Does not degrade	1. Nontoxic 2. Minimal signs of stenosis 3. Maintenance of airway patency 4. No granulation tissue found in lumen	1. The mesh provided stiffness against compression 2. Mechanical resistance against compression matched the native trachea	In Vivo with Dogs	End-to-End Anastomosis	All dogs survived and had an uneventful postoperative course.

### 3.8. Roadblocks Impacting Innovation

A major drawback of tissue engineering constructs lies in the time frame. With mucosalisation and epithelialisation taking significant time, the process is only hindered when applied to longer tracheal segments. With epithelial failure during neo-tracheal migration, an incomplete process results in cicatrix formation and without an epithelial barrier as protection, it opens a risk of bacterial infection, re-granulation, and potential restenosis [60]. With 3DP PCL rings, there were fibrotic deposits surrounding the luminal areas of the ring, which were implanted as a patch due to the acute and chronic inflammation resulting from its initial implantation. If significant, this could cause stenosis and require a re-operation. A necrotic process could follow an untreated fibrotic accumulation, leading to further infection and a substantial reduction in the cellular presence within the patch [15]. With chondrocyte-seeded fibrin/hyaluronan producing a successful partial reconstruction, there was a lack of neo-cartilage formation, which inevitably impacted the mechanics and structural integrity of the trachea leading to future collapse [61]. Inadequate mechanical strength alongside a short absorption time has made PLGA difficult to incorporate for tracheal reconstruction. Despite their high porosity allowing cell infiltration and neovascularization [62,63], PLGA is not considered for a long-term therapeutic effect. Due to the trachea being primarily sourced by the inferior thyroid artery and superior bronchial artery, there is a lack of arteriovenous vessels for anastomosis, which prevents the graft from utilising the tracheal blood supply. Without a supply of endothelial progenitor cells for neovascularization, graft necrosis from vascular ischemia and cell death will result [17]. Long-segment tracheal reconstruction faces the biggest challenge, with many animals dying from respiratory distress caused by airway stenosis [30]. Within paediatric situations, growth distortion can occur due to cartilage immaturity, which creates a growth disparity between the trachea and the graft, predisposing tracheal collapse. One limitation lies in the limited applicability of these findings to tracheal reconstruction in human subjects. Assessing these innovations in smaller animals, such as mice and rabbits, does not provide a comprehensive evaluation of all the clinical manifestations associated with larger animals or humans. Furthermore, as the size of the tracheal grafts and models increases, there is a potential rise in associated comorbidity, which cannot be precisely gauged or anticipated when conducting experiments on smaller creatures [35]. When utilising plates and other resorbing models, some test animals showed plate migration into the airway lumen through its displacement, which required an operation to remove the dislocated plate [40].

Tracheal epithelial harvesting is a difficult and time-consuming procedure that provokes unnecessary damage to the native trachea. The introduction of cells onto a decellularized graft could cause issues with vascularisation and epithelial growth production, and an allograft model could create an unpredictable reaction in the patient’s immune system causing a type IV hypersensitivity [44]. With all stem cells, questions arise of their clinical safety, as their tumorigenicity and risk of infection have not yet been fully established [49]. Their isolation requires invasive procedures that inflict pain on the patient and, with the advanced age of some donors, the therapeutic application of cell differentiation into functional tissue may be hindered [22].

## 4. Discussion

**Requirements for tracheobronchial reconstruction using synthetic material fabrication (including tracheal vascularization):**

Tracheal reconstruction through the innovations in synthetic material fabrication represents a promising approach to address complex tracheomalacia and stenotic conditions. Nonetheless, achieving success with these methodologies requires addressing formidable challenges. The trachea’s intricate anatomy—characterised by limited blood supply and a cylindrical framework enabling flexibility and stability—poses a significant hurdle for researchers. Replicating and regenerating the trachea, despite advancements in 3D printing and tissue engineering, remains a complex feat.

**Comments on the recent advances in 3D printing:**

Early research utilising 3D printed synthetic material in smaller animal models has demonstrated encouraging results. These findings lay the groundwork for further exploration and development. However, to ascertain the practicality and safety of these methods in human patients, transitioning from animal studies to comprehensive clinical trials is imperative. Clinical trials would offer crucial insights into the anatomo-physiological impact on the human body, elucidating short-term and long-term effects and the potential complications arising from implanted materials or cells.

**Comments on the recent advances in tissue engineering:**

Recent advancements in synthetic material fabrication using cell seeding have shown promise in addressing tracheal disorders. The fusion of these technologies has offered insights into potential solutions for challenging pathologies. Nevertheless, significant hurdles persist, necessitating a deeper understanding of how these approaches function in the context of human physiology. Rigorous clinical evaluations are pivotal to ensure their safety and efficacy in tracheal reconstruction procedures.

**Comments on animal applications:**

The application of synthetic material fabrication in animal studies has yielded encouraging results, laying the groundwork for potential advancements in tracheal reconstruction. However, the translation of these findings to human patients requires comprehensive clinical validation. Extensive animal studies provide a foundational understanding but necessitate further exploration and refinement through clinical trials to ascertain the practicality and safety of these techniques in human subjects.

**Comments on human applications:**

Although the potential benefits of synthetic material fabrication in tracheal reconstruction are promising, their widespread implementation faces numerous challenges. Navigating the complexities of tracheal anatomy and progressing toward robust clinical evaluation are critical. These innovative technologies hold promise in improving treatment options for individuals with tracheal disorders. Nonetheless, further research and stringent clinical validation are indispensable to ensure their safety and effectiveness in surgical practices.

## 5. Conclusions

The advancements in synthetic material fabrication have shown promise in the field of tracheal reconstruction, particularly in dealing with tracheomalacia and stenotic pathologies. These innovative approaches have demonstrated positive outcomes in initial studies, especially in smaller animal models. However, to fully realize the applicability of these developments, further research should progress toward clinical trials. These trials would enable a comprehensive assessment of the anatomical and physiological effects on the human body, providing a thorough evaluation of post-operative outcomes and potential complications related to the materials or cells implanted in the trachea. While challenges remain, the potential benefits of synthetic material fabrication in tracheal reconstruction offer hope for improved treatment options in the future.

## Data Availability

Data availability on request.
